# Characterization of the Complete Mitochondrial Genome of *Cerura menciana* and Comparison with Other Lepidopteran Insects

**DOI:** 10.1371/journal.pone.0132951

**Published:** 2015-08-26

**Authors:** Lishang Dai, Cen Qian, Congfen Zhang, Lei Wang, Guoqing Wei, Jun Li, Baojian Zhu, Chaoliang Liu

**Affiliations:** College of Life Science, Anhui Agricultural University, Anhui, Hefei, P.R. China; Institute of Plant Physiology and Ecology, CHINA

## Abstract

The complete mitochondrial genome (mitogenome) of *Cerura menciana* (Lepidoptera: Notodontidae) was sequenced and analyzed in this study. The mitogenome is a circular molecule of 15,369 bp, containing 13 protein-coding genes (PCGs), two ribosomal RNA (rRNA) genes, 22 transfer RNA (tRNA) genes and a A+T-rich region. The positive AT skew (0.031) indicated that more As than Ts were present. All PCGs were initiated by ATN codons, except for the cytochrome c oxidase subunit 1 (*cox1*) gene, which was initiated by CAG. Two of the 13 PCGs contained the incomplete termination codon T or TA, while the others were terminated with the stop codon TAA. The A+T-rich region was 372 bp in length and consisted of an ‘ATAGA’ motif followed by an 18 bp poly-T stretch, a microsatellite-like (AT)_8_ and a poly-A element upstream of the *trnM* gene. Results examining codon usage indicated that *Asn*, *Ile*, *Leu2*, *Lys*, *Tyr* and *Phe* were the six most frequently occurring amino acids, while *Cys* was the rarest. Phylogenetic relationships, analyzed based on the nucleotide sequences of the 13 PCGs from other insect mitogenomes, confirmed that *C*. *menciana* belongs to the Notodontidae family.

## Introduction

The insect mitochondrial DNA (mtDNA) is a circular DNA molecule, 14–19 kb in size [1]. It contains seven NADH dehydrogenase genes (*nad1-nad6* and *nad4L*), three cytochrome c oxidase genes (*cox1-cox3*), two ATPase genes (*atp6* and *atp8*), one cytochrome b (*cob*) gene, two ribosomal RNA genes (*rrnL* and *rrnS*), 22 transfer RNA (tRNA) genes and an adenine (A) + thymine (T)- rich region containing some initiation sites for transcription and replication of the genome [2,3]. MtDNA is maternally inherited and is subject to little if any sequence recombination, and is thus, useful for identifying species and characterizing population genetic structure and molecular evolution [4–7].

The order Lepidoptera contains more than 160,000 described species, classified into 45–48 superfamilies [8]. The superfamily Noctuoidea is the largest with about 42,400 species [4,9]. Despite this huge species diversity, information on the mitochondrial genome (mitogenome) of the Noctuoidea is very limited (Table 1). The moth, *Cerura menciana* (Notodontidae) is a pest of plants such as *Salix chaenomeloides* and *S*. *babylonica*, with two or three generations annually, distributed throughout northeastern China. Previous studies have investigated aspects of host preference and the natural enemies of *C*. *menciana* [10,11]. Characterization of the mitogenome of *C*. *menciana* will facilitate further insight into the evolutionary relationships of lepidopteran insects, especially in gene rearrangements. In this study, we characterize the complete mitogenome sequence of *C*. *menciana* and compared this with the mitogenome of other lepidopteran species.

**Table 1:** Details of the lepidopteran mitogenomes used in this study.

Subfamily	Family	Species	Size (bp)	Accession number	Reference
Noctuoidea	Noctuidae	*Spodoptera litura*	15,383	KF701043	[23]
		*Agrotis ipsilon*	15,377	KF163965	[37]
	Lymantriidae	*Lymantria dispar*	15,569	NC_012893	Unpublished
	Erebidae	*Hyphantria cunea*	15,481	GU592049	[38]
	Notodontidae	*Amata formosae*	15,463	KC513737	[4]
		*Ochrogaster lunifer*	15,593	AM946601	[5]
Bombycoidea	Bombycidae	*Phalera flavescens*	15,659	JF440342	[24]
		*C*. *menciana Moore*	15,369		This study
		*Bombyx mori*	15,643	NC_002355	Unpublished
		*Bombyx mandarina*	15,682	AY301620	[25]
	Saturniidae	*Actias selene*	15,236	NC_018133	[21]
		*Antheraea pernyi*	15,566	AY242996	[29]
		*Eriogyna pyretorum*	15,327	FJ685653	[1]
Pyraloidea	Crambidae	*Tyspanodes hypsalis*	15,329	NC_025569	[39]
	Pyralidae	*Lista haraldusalis*	15,213	NC_024535	[40]
Tortricoidea	Tortricidae	*Cydia pomonella*	15,253	JX407107	[41]
		*Grapholita dimorpha*	15,831	KJ671625	[42]
Gelechioidea	Oecophoridae	*Endrosis sarcitrella*	15,317	KJ508037	[43]
Papilionoidea	Papilionidae	*Luehdorfia taibai*	15,553	KC952673	[44]
	Nymphalidae	*Apatura ilia*	15,242	NC_016062	[45]

## Materials and Methods

### Experimental insects and DNA extraction

*C*. *menciana* larvae of both sexes were collected from willow trees within the campus of Anhui Agricultural University, Hefei city, China. The owner of the land gave permission to conduct the study on this site and the work did not involve endangered or protected species. Total genomic DNA was extracted from larvae using the Aidlab Genomic DNA Extraction Kit (Aidlab Co., Beijing, China) according to the manufacturer's instructions. DNA was examined on a 1% agarose gel and used for PCR amplification of the complete mitogenome.

### PCR amplification, cloning and sequencing

To amplify the whole mitogenome of *C*. *menciana*, we designed thirteen pairs of universal primers according to published mitogenomes from other Notodontidae insects, which were then synthesized by SangonBiotech Co., Shanghai, China (Table 2). All PCRs were performed in a 50 μL reaction volume, including 35 μL sterilized distilled water, 5 μL 10 × Taq buffer (Mg2+ plus), 4 μL dNTP (25 mM), 1.5 μL DNA, 2 μL each primer (10 μM) and 0.5 μL (1 unit) Taq (Aidlab Co., Beijing, China). The PCR was performed under the following conditions: an initial denaturation at 94°C for 4 min followed by 35 cycles of 30 s at 94°C, 40 s at 49–58°C (depending on primer combination), 1–3 min (depending on putative length of the fragments) at 72°C, and a final extension step of 72°C for 10 min.

**Table 2:** Details of the primers used to amplify the mitogenome of *C*. *menciana*.

Primer pair	Primer sequence (5’ →3’)
F1	TAAAAATAAGCTAAATTTAAGCTT
R1	TATTAAAATTGCAAATTTTAAGGA
F2	AAACTAATAATCTTCAAAATTAT
R2	AAAATAATTTGTTCTATTAAAG
F3	ATTCTATATTTCTTGAAATATTAT
R3	CATAAATTATAAATCTTAATCATA
F4	TGAAAATGATAAGTAATTTATTT
R4	AATATTAATGGAATTTAACCACTA
F5	TAAGCTGCTAACTTAATTTTTAGT
R5	CCTGTTTCAGCTTTAGTTCATTC
F6	CCTAATTGTCTTAAAGTAGATAA
R6	TGCTTATTCTTCTGTAGCTCATAT
F7	TAATGTATAATCTTCGTCTATGTAA
R7	ATCAATAATCTCCAAAATTATTAT
F8	ACTTTAAAAACTTCAAAGAAAAA
R8	TCATAATAAATTCCTCGTCCAATAT
F9	GTAAATTATGGTTGATTAATTCG
R9	TGATCTTCAAATTCTAATTATGC
F10	CCGAAACTAACTCTCTCTCACCT
R10	CTTACATGATCTGAGTTCAAACCG
F11	CGTTCTAATAAAGTTAAATAAGCA
R11	AATATGTACATATTGCCCGTCGCT
F12	TCTAGAAACACTTTCCAGTACCTC
R12	AATTTTAAATTATTAGGTGAAATT
F13	TAATAGGGTATCTAATCCTAGTT
R13	ACTTAATTTATCCTATCAGAATAA

PCR products were separated on a 1% agarose gel and purified using a DNA gel extraction kit (Transgen Co., Beijing, China). The purified PCR fragments were ligated into the T-vector (TaKaRa Co., Dalian, China) and then transformed into *Escherichia coli* DH5α. Recombinants were cultured overnight at 37°C in Luria-Bertani (LB) solid medium containing Ampicillin (AMP), isopropylthiogalactoside (IPTG) and 5-bromo-4-chloro-3-indolyl-D-galactopyranoside (X-Gal). White colonies carrying insert DNA were selected, grown overnight in liquid media, and then sequenced at least three times by Invitrogen Co. Ltd. (Shanghai, China).

### Sequence assembly and gene annotation

The final consensus sequence of the mtDNA of *C*. *menciana* was performed using the SeqMan II program from the Lasergene software package (DNAStar Inc., Madison, USA). Sequence annotation was performed using the online blast tools in NCBI website (http://blast.ncbi.nlm.nih.gov/Blast).

The nucleotide sequences of the PCGs were initially translated into putative proteins on the basis of the invertebrate mtDNA genetic code. These exact initiation and termination codons were identified in ClustalX version 2.0 using reference sequences from other lepidopteran insects. To describe the base composition of nucleotide sequences, we calculated composition skewness as described by Junqueira [12]: AT skew = [A−T]/[A+T], GC skew = [G−C]/[G+C]. The Relative Synonymous Codon Usage (RSCU) values were calculated using MEGA 5.0 [13]. The overlapping regions and intergenic spacers between genes were counted manually.

The tRNA genes were verified using either program tRNAscan-SE Search with the default settings [14] or by manually identifying sequences with the appropriate anticodon capable of folding into the typical cloverleaf secondary structure. Tandem repeats in the A+T-rich region were found with the Tandem Repeats Finder program (http://tandem.bu.edu/trf/trf.html) [15].

### Phylogenetic analysis

Twenty lepidopteran mitogenomes were downloaded from GenBank to illustrate the phylogenetic relationships among lepidoptera insects. The mitogenomes of *Drosophila incompta* (NC_025936) [16] and *Anopheles gambiae* (NC_002084) [17] were downloaded and used as outgroups. The multiple alignments of the 13 PCG concatenated nucleotide sequences of these lepidopteran mitogenomes was conducted using ClustalX version 2.0. The phylogenetic analysis was performed using Maximum Likelihood (ML) method with the MEGA 5.0 program [13].

## Results and Discussion

### Genome structure, organization and composition

**Fig 1:**
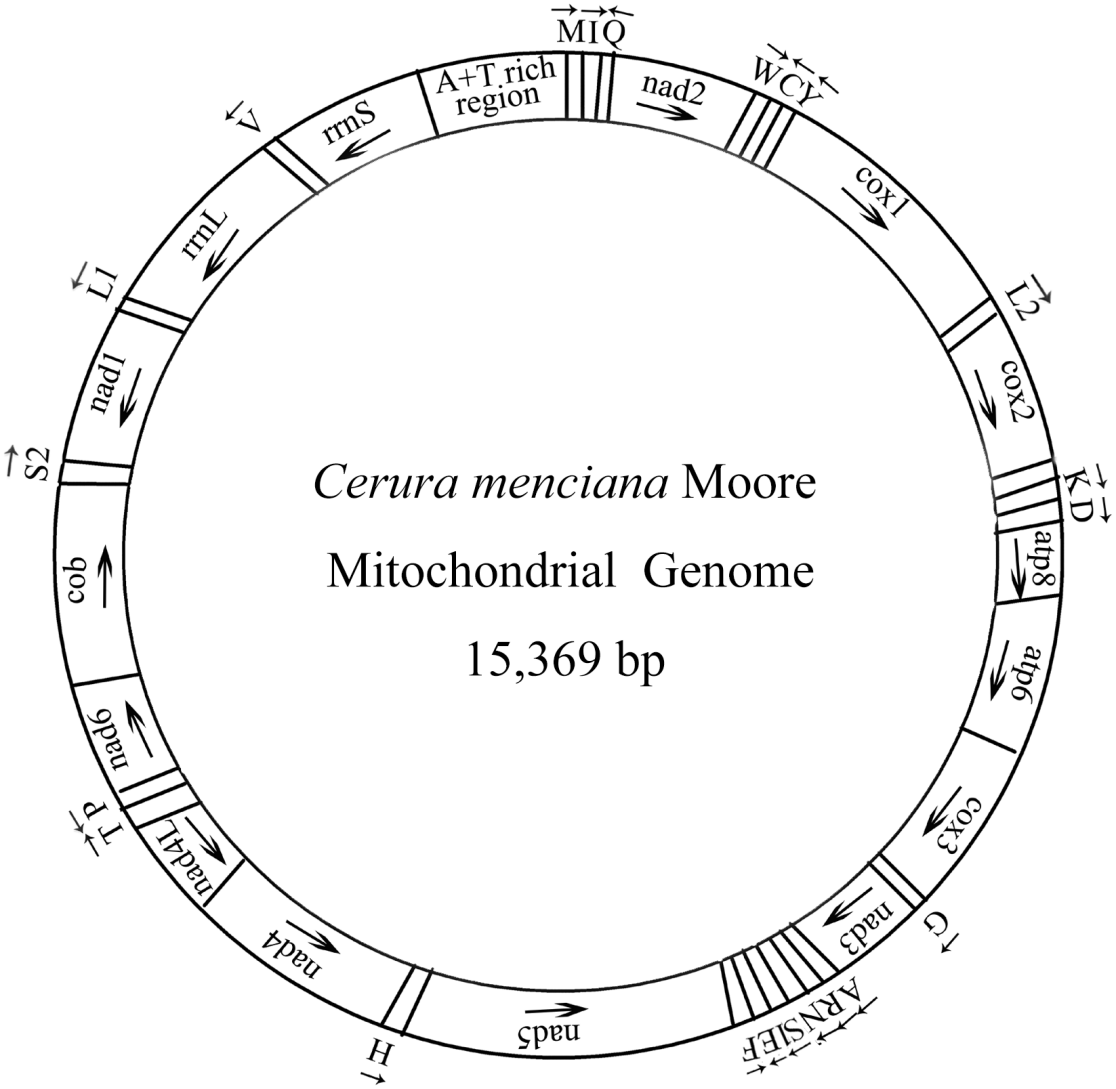
Map of the mitogenome of *C*. *menciana*. The tRNA genes are labeled according to the IUPAC-IUB single-letter amino acids: *cox1*, *cox2* and *cox3* refer to the cytochrome c oxidase subunits; *cob* refers to cytochrome b; *nad1-nad6* refer to NADH dehydrogenase components; *rrnL* and *rrnS* refer to ribosomal RNAs. Gene named above the bar are located on major strand, while the others are located on minor strand.

We report that the complete mitogenome of *C*. *menciana* is a circular molecule of 15,369 bp in size (Fig 1). This is within the range for similar organisms: 15,236 in *Actias selene* to 15,831 in *Grapholita dimorpha*. The mitogenome contains the typical gene content observed in metazoan mitogenomes: containing 22 tRNA genes, 13 PCGs (*nad1-6*, *nad4L*, *cox1-3*, *cob*, *atp6* and *atp8*), two rRNAs (*rrnS* and *rrnL*), and an A+T-rich region (Table 3). Gene order and orientation of *C*. *menciana* was *trnM-trnI-trnQ*, which differs from the ancestral order *trnI-trnQ-trnM* [18]. The nucleotide composition is highly A+T biased (A: 41.28%, T: 38.78%, G: 7.61%, C: 12.32%; Table 4). This is within the range for similar species (A+T bias of 77.84% in *Ochrogaster lunifer* and 81.59% in Chinese *Bombyx mandarina*). The positive AT skew we observed (0.031) indicates the occurrence of more As than Ts, similar to other lepidopterans, including *Lymantria dispar* (0.016), *Hyphantria cunea* (0.010), *O*. *lunifer* (0.030), Chinese *B*. *mandarina* (0.057). Lepidopteran mitogenomes exhibit negative GC skewness ranging in size from -0.172 to -0.318 (Table 4). The GC skewness of *C*. *menciana* mitogenome rRNA was far lower than this range (−0.416; Table 4), indicating a particularly heavy bias toward Cs and against Gs in the rRNA. This phenomenon is known from other lepidopteran insects [1,19–21].

**Table 3:** Summary results for characteristics of the mitogenome of *C*. *menciana*.

Gene	Direction	Location	Size	Anticodon	Start codon	Stop codon	Intergenic Nucleotides *
*trnM*	F	1–68	68	CAT	—	—	0
*trnI*	F	69–134	66	GAT	—	—	-3
*trnQ*	R	132–200	69	TTG	—	—	57
*nad2*	F	258–1271	1014	—	ATT	TAA	17
*trnW*	F	1289–1361	73	TCA	—	—	-8
*trnC*	R	1354–1422	69	GCA	—	—	2
*trnY*	R	1425–1490	66	GTA	—	—	5
*cox1*	F	1496–3026	1531	—	CGA	T	0
*trnL2(UUR)*	F	3027–3093	67	TAA	—	—	0
*cox2*	F	3094–3775	682	—	ATG	T	0
*trnK*	F	3776–3846	71	CTT	—	—	-1
*trnD*	F	3846–3911	67	GTC	—	—	0
*atp8*	F	3912–4070	159	—	ATC	TAA	-7
*atp6*	F	4064–4741	678	—	ATG	TAA	-1
*cox3*	F	4741–5529	789	—	ATG	TAA	2
*trnG*	F	5532–5597	66	TCC	—	—	0
*nad3*	F	5598–5951	354	—	ATC	TAA	53
*trnA*	F	6005–6074	70	TGC	—	—	-1
*trnR*	F	6074–6137	64	TCG	—	—	3
*trnN*	F	6141–6205	65	GTT	—	—	1
*trnS1(AGN)*	F	6207–6276	70	GCT	—	—	1
*trnE*	F	6278–6345	68	TTC	—	—	-2
*trnF*	R	6344–6410	67	GAA	—	—	-2
*nad5*	R	6409–8152	1744	—	ATT	TAA	-2
*trnH*	R	8151–8216	66	GTG	—	—	-2
*nad4*	R	8215–9557	1339	—	ATA	TA	-4
*nad4L*	R	9554–9841	288	—	ATT	TAA	14
*trnT*	F	9856–9920	65	TGT	—	—	0
*trnP*	R	9921–9985	65	TGG	—	—	8
*nad6*	F	9994–10,524	531	—	ATT	TAA	13
*cob*	F	10,539–11,687	1149	—	ATG	TAA	4
*trnS2(UCN)*	F	11,692–11,758	67	TGA	—	—	18
*nad1*	R	11,777–12,716	940	—	ATT	TAA	7
*trnL1(CUN)*	R	12,724–12,794	71	TAG	—	—	0
*rrnL*	R	12,795–14,152	1358	—	—	—	0
*trnV*	R	14,153–14,219	67	TAC	—	—	0
*rrnS*	R	14,220–14,998	779	—	—	—	0
A+T-rich Region		14,999–15,370	372	—	—	—	—

**Table 4:** Composition and skewness in different Lepidopteran mitogenomes.

Species	Size (bp)	A%	G%	T%	C%	A+T %	ATskewness	GCskewness
**Whole genome**								
***C*. *menciana***	**15,369**	**41.28**	**7.61**	**38.78**	**12.32**	**80.06**	**0.031**	**-0.236**
*A*. *ipsilon*	15,377	40.38	7.71	40.87	11.04	81.25	-0.006	-0.178
*L*. *dispar*	15,569	40.58	7.57	39.30	12.55	79.88	0.016	-0.248
*H*. *cunea*	15,481	40.58	7.55	39.81	12.06	80.39	0.010	-0.230
*A*. *formosae*	15,453	38.67	7.53	40.83	12.98	79.49	-0.027	-0.266
*O*. *lunifer*	15,593	40.09	7.56	37.75	14.60	77.84	0.030	-0.318
*P*. *flavescens*	15,659	40.07	7.87	40.80	11.26	80.87	-0.009	-0.177
*B*. *mandarina*	15,682	43.11	7.40	38.48	11.01	81.59	0.057	-0.196
*A*. *selene*	15,236	38.54	8.05	40.37	13.03	78.91	-0.023	-0.236
*A*. *pernyi*	15,566	39.22	7.77	40.94	12.07	80.16	-0.021	-0.216
*E*. *pyretorum*	15,327	39.17	7.63	41.65	11.55	80.82	-0.031	-0.204
*T*. *hypsalis*	15,329	40.00	7.67	41.42	10.92	81.41	-0.017	-0.175
*L*. *haraldusalis*	15,213	40.47	7.66	41.04	10.83	81.52	-0.007	-0.172
*C*. *pomonella*	15,253	39.92	7.88	40.21	11.99	80.13	-0.004	-0.207
*G*. *dimorpha*	15,831	39.99	7.77	40.85	11.39	80.84	-0.011	-0.189
*L*. *taibai*	15,553	40.37	7.39	41.10	11.14	81.46	-0.009	-0.202
*A*. *ilia*	15,242	39.77	7.75	40.68	11.80	80.45	-0.011	-0.207
**PCG**								
***C*. *menciana***	**11,190**	**40.68**	**8.40**	**37.72**	**13.20**	**78.42**	**0.038**	**-0.222**
*A*. *ipsilon*	11,226	39.69	8.44	40.14	11.72	79.83	-0.006	-0.163
*L*. *dispar*	11,227	39.67	8.44	38.16	13.73	77.83	0.019	-0.239
*H*. *cunea*	11,198	39.98	8.35	38.61	13.06	78.59	0.017	-0.220
*A*. *formosae*	11,217	38.18	8.28	39.62	13.92	77.80	-0.019	-0.254
*O*. *lunifer*	11,266	32.47	12.08	43.26	12.19	75.73	-0.142	-0.004
*P*. *flavescens*	11,206	39.40	8.90	39.56	12.15	78.96	-0.002	-0.154
*B*. *mandarina*	11,196	42.83	8.26	37.04	11.87	79.87	0.072	-0.179
*A*. *selene*	11,231	37.93	8.74	39.44	13.89	77.37	-0.020	-0.228
*A*. *pernyi*	11,204	39.22	7.77	40.94	12.07	80.16	-0.021	-0.216
*E*. *pyretorum*	11,228	33.18	10.50	46.23	10.09	79.41	-0.164	0.020
*T*. *hypsalis*	11,188	39.31	8.46	40.66	11.57	79.97	-0.017	-0.155
*L*. *haraldusalis*	11,193	39.88	8.47	40.16	11.49	80.04	-0.003	-0.151
*C*. *pomonella*	11,199	39.55	8.69	39.00	12.76	78.55	0.007	-0.190
*G*. *dimorpha*	11,232	39.51	8.81	39.18	12.49	78.69	0.004	-0.173
*L*. *taibai*	11,178	39.56	8.26	40.18	12.01	79.74	-0.008	-0.185
*A*. *ilia*	11,148	39.41	8.41	39.49	12.69	78.89	-0.001	-0.203
**tRNA**								
***C*. *menciana***	**1472**	**42.12**	**7.81**	**40.01**	**10.05**	**82.13**	**0.026**	**-0.125**
*A*. *ipsilon*	1465	41.23	8.12	40.48	10.17	81.71	0.014	-0.112
*L*. *dispar*	1459	41.60	7.95	39.48	10.97	81.08	0.026	-0.160
*H*. *cunea*	1463	41.83	7.86	39.99	10.32	81.82	0.022	-0.135
*A*. *formosae*	1457	40.43	7.96	40.36	11.26	80.78	0.001	-0.172
*O*. *lunifer*	1666	41.78	7.32	39.86	11.04	81.63	0.023	-0.202
*P*. *flavescens*	1474	41.66	7.80	40.64	9.91	82.29	0.012	-0.119
*B*. *mandarina*	1472	41.78	7.81	39.95	10.46	81.73	0.022	-0.145
*A*. *selene*	1459	40.37	8.16	40.23	11.24	80.60	0.002	-0.159
*A*. *pernyi*	1459	39.22	7.77	40.94	12.07	80.16	-0.021	-0.216
*E*. *pyretorum*	1424	42.59	10.61	39.35	7.45	81.94	0.039	0.174
*T*. *hypsalis*	1456	40.73	7.90	41.35	10.03	82.07	-0.008	-0.119
*L*. *haraldusalis*	1451	41.08	7.86	41.42	9.65	82.49	-0.004	-0.102
*C*. *pomonella*	1451	41.14	7.93	40.32	10.61	81.46	0.010	-0.145
*G*. *dimorpha*	1451	41.01	8.06	40.52	10.41	81.53	0.006	-0.127
*L*. *taibai*	1440	41.39	7.85	40.90	9.86	82.29	0.006	-0.113
*A*. *ilia*	1433	40.61	8.30	40.96	10.12	81.58	-0.004	-0.099
**rRNA**								
***C*. *menciana***	**2137**	**42.82**	**4.73**	**40.99**	**11.46**	**83.81**	**0.022**	**-0.416**
*A*. *ipsilon*	2162	41.58	5.00	43.57	9.85	85.15	-0.023	-0.327
*L*. *dispar*	2150	42.79	4.79	41.81	10.60	84.60	0.012	-0.377
*H*. *cunea*	2234	42.08	4.92	42.75	10.25	84.83	-0.008	-0.351
*A*. *formosae*	2163	38.93	4.72	44.85	11.51	83.77	-0.071	-0.418
*O*. *lunifer*	2157	41.96	4.82	40.19	13.03	82.15	0.022	-0.460
*P*. *flavescens*	2198	41.31	4.73	44.04	9.92	85.35	-0.032	-0.354
*B*. *mandarina*	2134	43.86	4.78	41.05	10.31	84.91	0.028	-0.366
*A*. *selene*	2126	39.93	4.99	43.79	11.29	83.73	-0.046	-0.387
*A*. *pernyi*	2144	39.22	7.77	40.94	12.07	80.16	-0.021	-0.216
*E*. *pyretorum*	2116	41.16	4.82	43.38	10.63	84.55	-0.026	-0.376
*T*. *hypsalis*	2156	42.02	4.92	43.09	9.97	85.11	-0.013	-0.339
*L*. *haraldusalis*	2121	42.20	4.67	43.33	9.81	85.53	-0.013	-0.355
*C*. *pomonella*	2147	40.48	5.03	43.92	10.57	84.40	-0.041	-0.355
*G*. *dimorpha*	2181	41.13	4.95	43.83	10.09	84.96	-0.032	-0.342
*L*. *taibai*	1805	42.16	5.37	41.83	10.64	83.99	0.004	-0.329
*A*. *ilia*	2109	40.11	4.98	44.86	10.05	84.97	-0.056	-0.337
**A+T-rich region**								
***C*. *menciana***	**372**	**44.35**	**2.42**	**50.00**	**3.23**	**94.35**	**-0.060**	**-0.143**
*A*. *ipsilon*	332	46.08	1.51	48.80	3.61	94.88	-0.029	-0.410
*L*. *dispar*	435	40.58	7.57	39.30	12.55	79.88	0.016	-0.248
*H*. *cunea*	357	45.66	1.12	49.30	3.92	94.96	-0.038	-0.556
*A*. *formosae*	482	42.95	2.90	49.79	4.36	92.74	-0.074	-0.201
*O*. *lunifer*	319	44.5	1.6	48.9	5.0	93.4	-0.047	-0.524
*P*. *flavescens*	541	42.14	2.22	49.72	5.91	91.87	-0.083	-0.454
*B*. *mandarina*	484	46.49	2.69	47.93	2.89	94.42	-0.015	-0.036
*A*. *selene*	339	43.07	5.90	44.84	6.19	87.91	-0.020	-0.024
*A*. *pernyi*	552	39.22	7.77	40.94	12.07	80.16	-0.021	-0.216
*E*. *pyretorum*	358	42.18	2.51	50.00	5.31	92.18	-0.085	-0.358
*T*. *hypsalis*	350	43.43	1.14	52.00	3.43	95.43	-0.090	-0.501
*L*. *haraldusalis*	310	45.81	0.97	50.32	2.90	96.13	-0.047	-0.499
*C*. *pomonella*	351	43.30	1.14	52.42	3.13	95.73	-0.095	-0.466
*G*. *dimorpha*	848	41.63	1.30	54.83	2.24	96.46	-0.137	-0.266
*L*. *taibai*	939	45.15	1.70	49.41	3.73	94.57	-0.045	-0.374
*A*. *ilia*	403	42.93	3.23	49.63	4.22	92.56	-0.072	-0.133

### Protein-coding genes and codon usage

We found that the 13 Protein-Coding Genes of *C*. *menciana* were 11,190 bp in length and accounted for 72.81% of the whole mitochondrial genome. Nine of these PCGs (*nad2*, *cox1*, *cox2*, *atp8*, *atp6*, *cox3*, *nad3*, *nad6* and *cob*) were coded by the H-strand, while the remaining four PCGs (*nad5*, *nad4*, *nad4L* and *nad1*) were coded by the L-strand. The AT skew was positive (0.038) indicating the occurrence of more As than Ts. All PCGs started with the canonical putative start codons ATN except for the *cox1* gene which started with CGA instead, similar to other lepidopterans [22,23]. Ten genes shared complete termination codon TAA, while three genes used incomplete stop codons (a single T for *cox1* and *cox2*, TA for *nad4*). The single T as a stop codon for *cox1* and *cox2* has been reported in the majority of the sequenced lepidopteran mitogenomes, and even in some mammalian mitochondrial genes [20,22].

**Fig 2:**
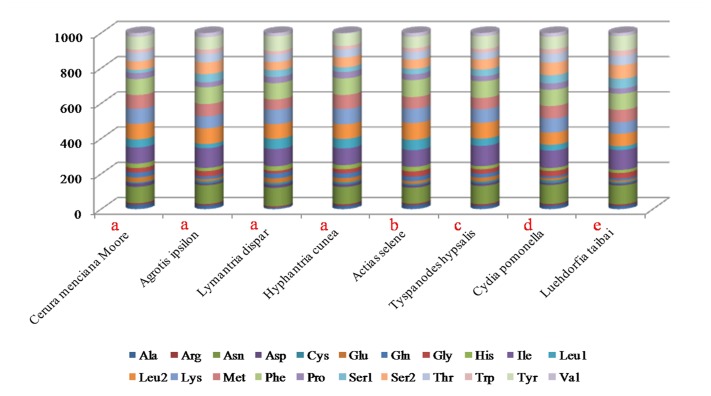
Comparison of codon usage within the mitochondrial genome of members of the Lepidoptera. Lowercase letters (a, b, c, d and e) above species name represent the superfamily to which the species belongs (a: Noctuoidea, b: Bombycoidea, c: Pyraloidea, d: Tortricoidea, e: Papilionoidea).

**Fig 3:**
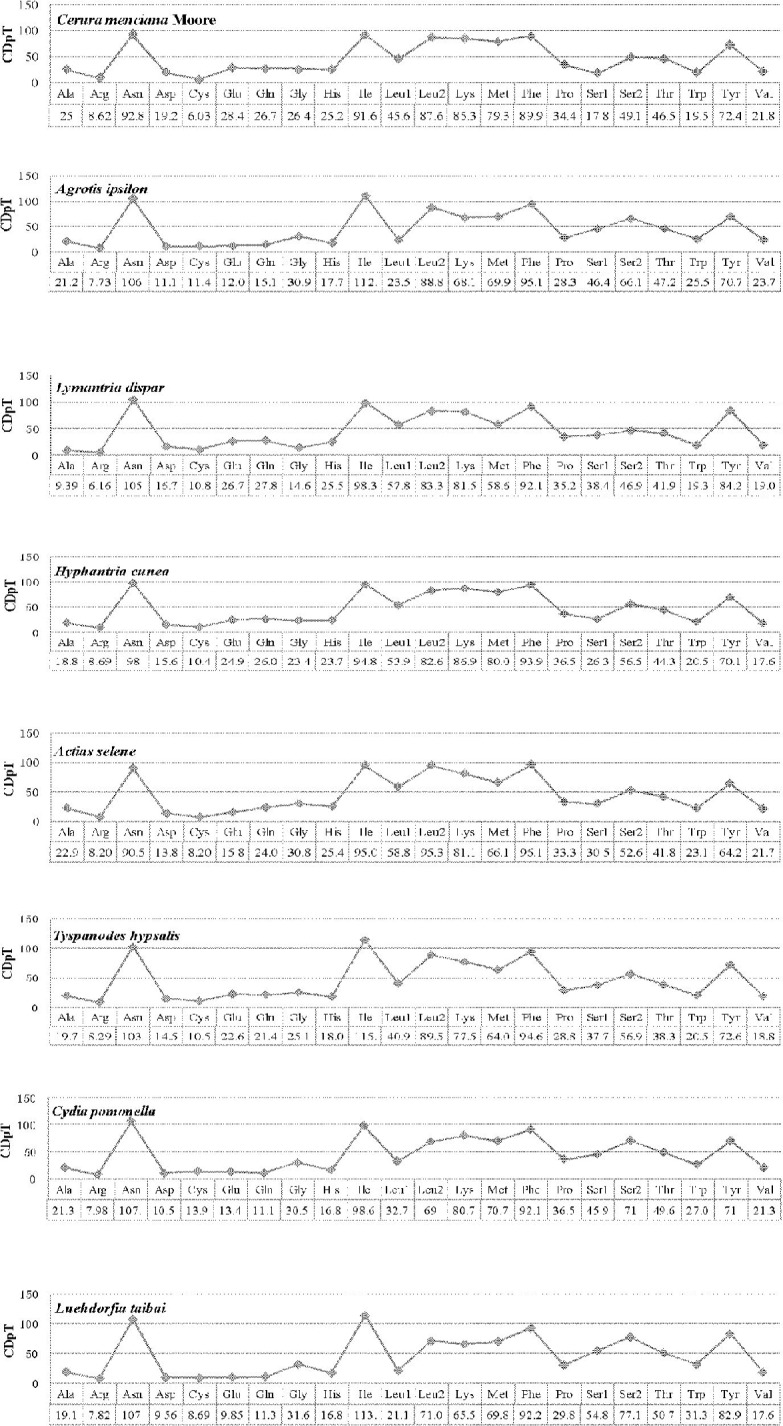
Codon distribution in members of the Lepidoptera. CDspT = codons per thousand codons.

**Fig 4:**
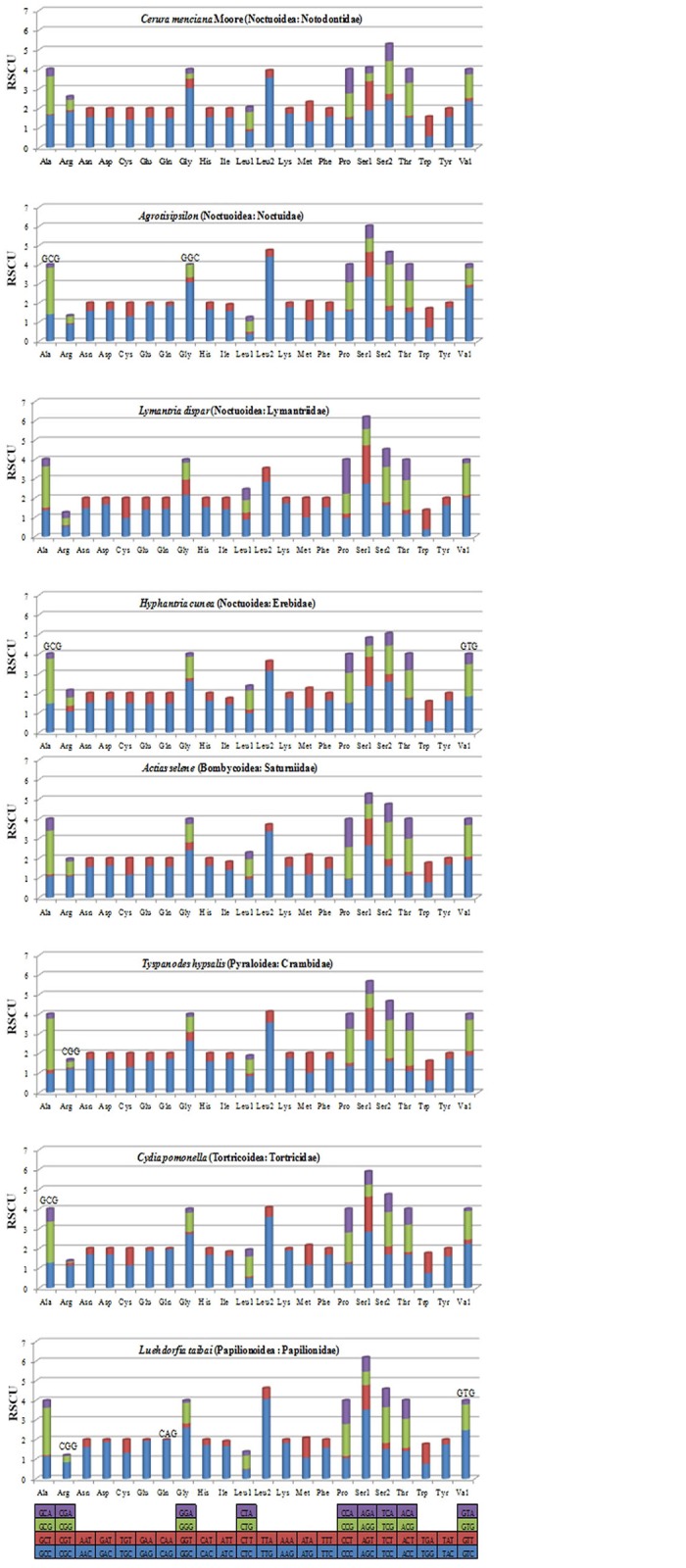
The Relative Synonymous Codon Usage (RSCU) of the mitochondrial genome of five superfamilies in the Lepidoptera. Codon families are plotted on the X axis. Codons indicated above the bar are not present in the mitogenome.

A comparison of the codon usage of eight mitochondrial genomes from the Lepidoptera reveals they are divided into five superfamilies: four species belonging to Noctuoidea, and four belonging to Bombycoidea, Pyraloidea, Tortricoidea, and Papilionoidea (Fig 2). Our results indicated that *Asn*, *Ile*, *Leu2*, *Lys*, *Tyr* and *Phe* were the six most frequently present amino acids, while *Cys* was rare. Codon distributions of four species in Noctuoidea are consistency and each amino acid has equal content in different species (Fig 3). All codons were present in the PCGs of the *C*. *menciana* mitogenome (Fig 4). This was similar to *L*. *dispar*, *A*. *selene* and *Tyspanodes hypsalis*, but differed from *A*. *ipsilon*, *H*. *cunea*, *C*. *pomonella* and *Luehdorfia taibai*, which lacked the codons GCG&GGC, GCG&GTG, GCG, CGG&CAG&GTG, respectively. Codons with a high GC content are abandoned in other some lepidopteran insects [4,24].

### Ribosomal RNA and transfer RNA genes

The *rrnL* and *rrnS* gene in *C*. *menciana* were located between *trnL1* (CUN) and *trnV*, and between *trnV* and the A+T-rich region, respectively. The *rrnL* was 1358 bp while *rrnS* was 779 bp. The A+T content of the two rRNA genes totaled 83.81%, which is within the previously range (80.16% in *Antheraea pernyi* to 85.53% in *Lista haraldusalis;* Table 3). The AT skew was positive (0.022), while the GC skew was negative (-0.416), similar to that reported for other sequenced lepidopteran mitogenomes [5,25].

**Fig 5:**
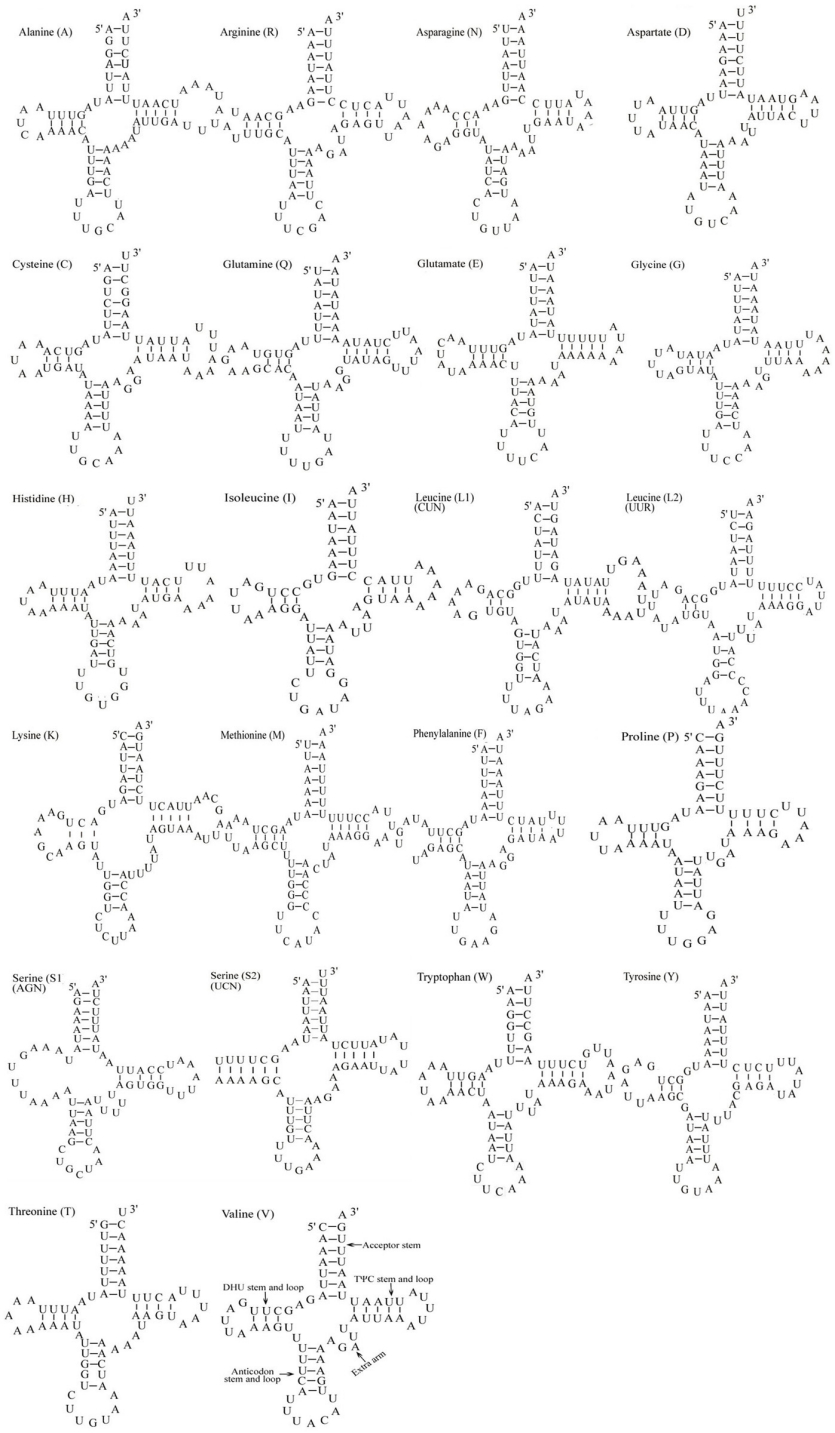
Putative secondary structures of the 22 tRNA genes of the *C*. *menciana* mitogenome.

The *C*. *menciana* mitogenome harbored 22 tRNA genes, ranging from 64 bp (*trnR*) to 73 bp (*trnW*). Fourteen genes were encoded on the H-strand with the rest on the L-strand (Table 3). The tRNA genes were also highly A+T biased (82.13%) and exhibited positive AT-skew (0.026; Table 4). All the tRNAs could be folded into the expected secondary cloverleaf structures except the *trnS1* (AGN) gene (Fig 5). In the *trnS1* (AGN) gene; its dihydrouridine (DHU) arm simply forms a loop, as is often found in several other insect mitogenomes [26–28]. Ten unmatched base pairs of G-U occurred in *C*. *menciana* mitochondrial tRNA genes. In addition, the *trnA* contained a U-U mismatch in the acceptor stem. All of mismatches were located in the acceptor, DHU and anticodon stems. The mismatches were scattered among 10 of the 22 *C*. *menciana* tRNA genes, including *trnA*, *trnC*, *trnQ*, *trnG*, *trnL1* (*CUN*), *trnL2* (*UUR*), *trnF*, *trnP*, *trnS1* (*AGN*) and *trnV* (Fig 5). All of the secondary structures were drawn by the RNAstructure program.

### Overlapping and intergenic spacer regions

Eleven overlapping sequences with a total length of 33 bp were identified in the *C*. *menciana* mitogenome. These sequences varied in length from 1 bp to 8 bp with the longest overlapping region present between *trnW* and *trnC* (Table 3). Other overlap regions included 7 bp between *atp8* and *atp6*, 4 bp between *nad4* and *nad4L*, 3 bp between the *trnI* and *trnQ*, with all other overlapping sequences shorter than 3 bp (Table 3). The 7-bp overlap between *atp8* and *atp6* is common in many Lepidoptera mitogenomes [29,30].

**Fig 6:**
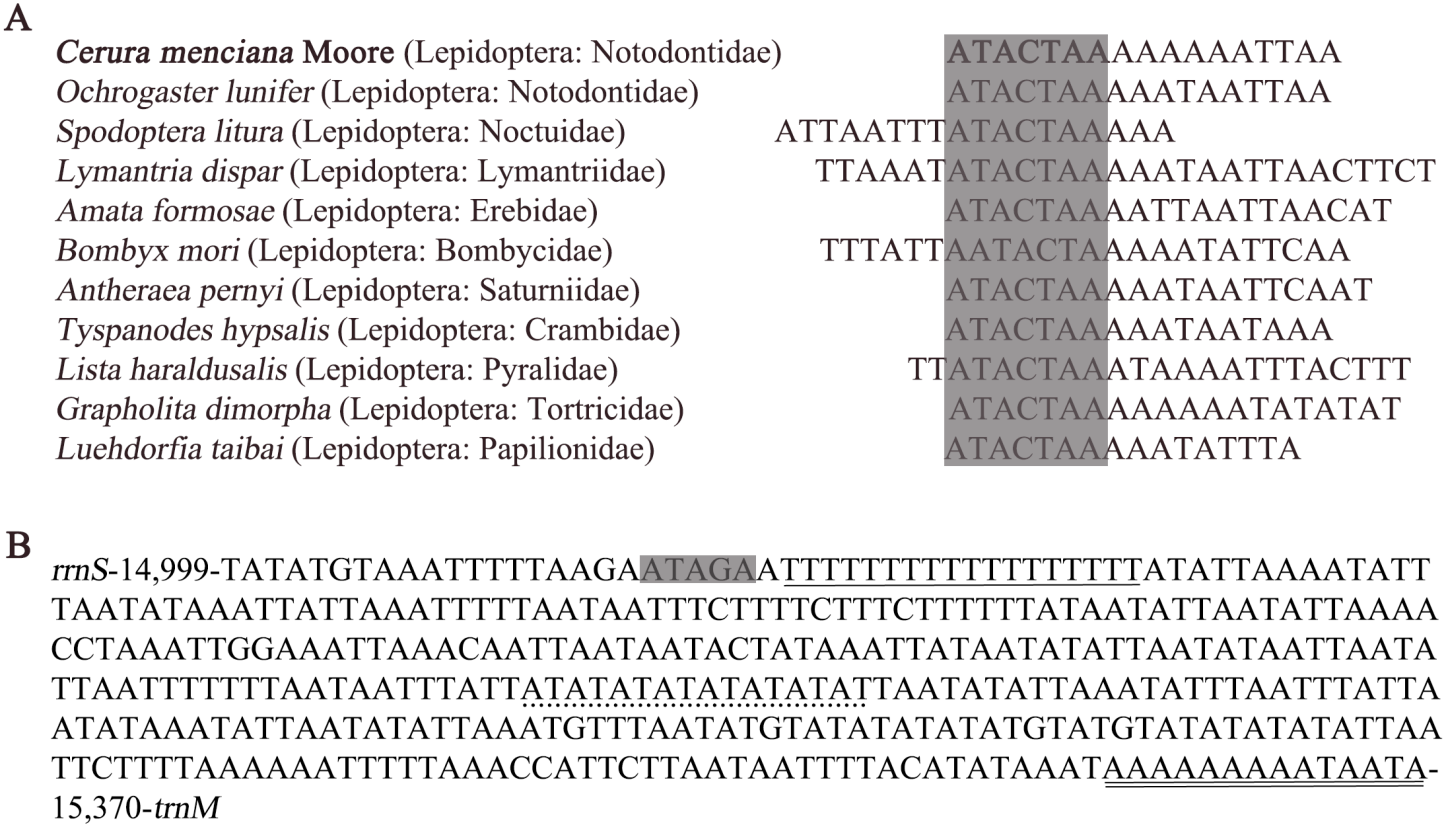
(A) Alignment of the intergenic spacer region between *trnS2 (UCN)* and *ND1* of several Lepidopteran insects. The shaded ‘ATACTAA’ motif is conserved across the Lepidoptera order. (B) Features present in the A+T-rich region of *C*. *menciana*. The sequence is shown in the reverse strand. The ATATG motif is shaded. The poly-T stretch is underlined while the poly-A stretch is double underlined. The single microsatellite T/A repeat sequence is indicated by dotted underlining.

The intergenic spacers of *C*. *menciana* mitogenomes, spread over 15 regions and ranged in size from 1 bp to 57 bp with a total length of 205 bp. The longest spacer (57 bp) was extremely A+T rich and occurred between *trnQ* and *nad2*. Intergenic spacers in *C*. *menciana* were shorter than those in *O*. *lunifer* (371 bp over 20 regions) but longer than those in *A*. *selene* (137 bp over 13 regions) [5,21]. The 18 bp spacer region between *trnS2* (*UCN*) and *nad1* contained the ‘ATACTAA’ motif. This 7 bp motif is a common feature amongst the 11 species of different families we selected, indicating that this region is conserved and present in most insect mtDNAs (Fig 6A).

### The A+T-rich region

The 372 bp (14,999–15,370 nt) A+T-rich region was located between the *rrnS* and *trnM* genes. This region is longer in *C*. *menciana* than in *A*. *ipsilon* (332 bp), *H*. *cunea* (357 bp), *A*. *selene* (339 bp), *O*. *lunifer* (319 bp), *Eriogyna pyretorum* (358 bp), *T*. *hypsalis* (350 bp), *L*. *haraldusalis* (310 bp) and *Cydia pomonella* (351 bp), but shorter than *L*. *dispar* (435 bp), *Amata formosae* (482 bp), *Phalera flavescens* (541 bp), *A*. *pernyi* (552 bp) and *Apatura ilia* (403 bp). The A+T-rich region harbors the highest A+T content (94.35%), most negative AT skew (-0.060) and most negative GC skew (-0.143). The presence of multiple tandem repeat elements has been reported to be a characteristic of the insect A+T-rich region [31]. For example, in *M*. *separate*, the A+T-rich region contains a duplicate 51 bp repeat element that occurs twice [8], while in *Cnaphalocrocis medinalis* there is a duplicated 25 bp repeat element and in *Chilo suppressalis* a duplicated 31 bp repeat element [32]. We found no conspicuous long repeats in the A+T-rich region of *C*. *menciana*. We did find several short repeating sequences scattered throughout the entire region, including the motif ‘ATAGA’ followed by an 18 bp poly-T stretch, a microsatellite-like (AT)_8_ and a poly-A element upstream of *trnM* gene (Fig 6B). These sequences are similar to those found in the genomes of other lepidopteran insects [21,33–35]. In addition, the presence of extra tRNA-like structures in the A+T-rich region has been reported in the lepidopteran insects, such as Chinese *B*. *mandarina* [31]. In this study, however, we did not detect a tRNA-like structure in the *C*. *menciana* A+T-rich region.

### Phylogenetic relationships

**Fig 7:**
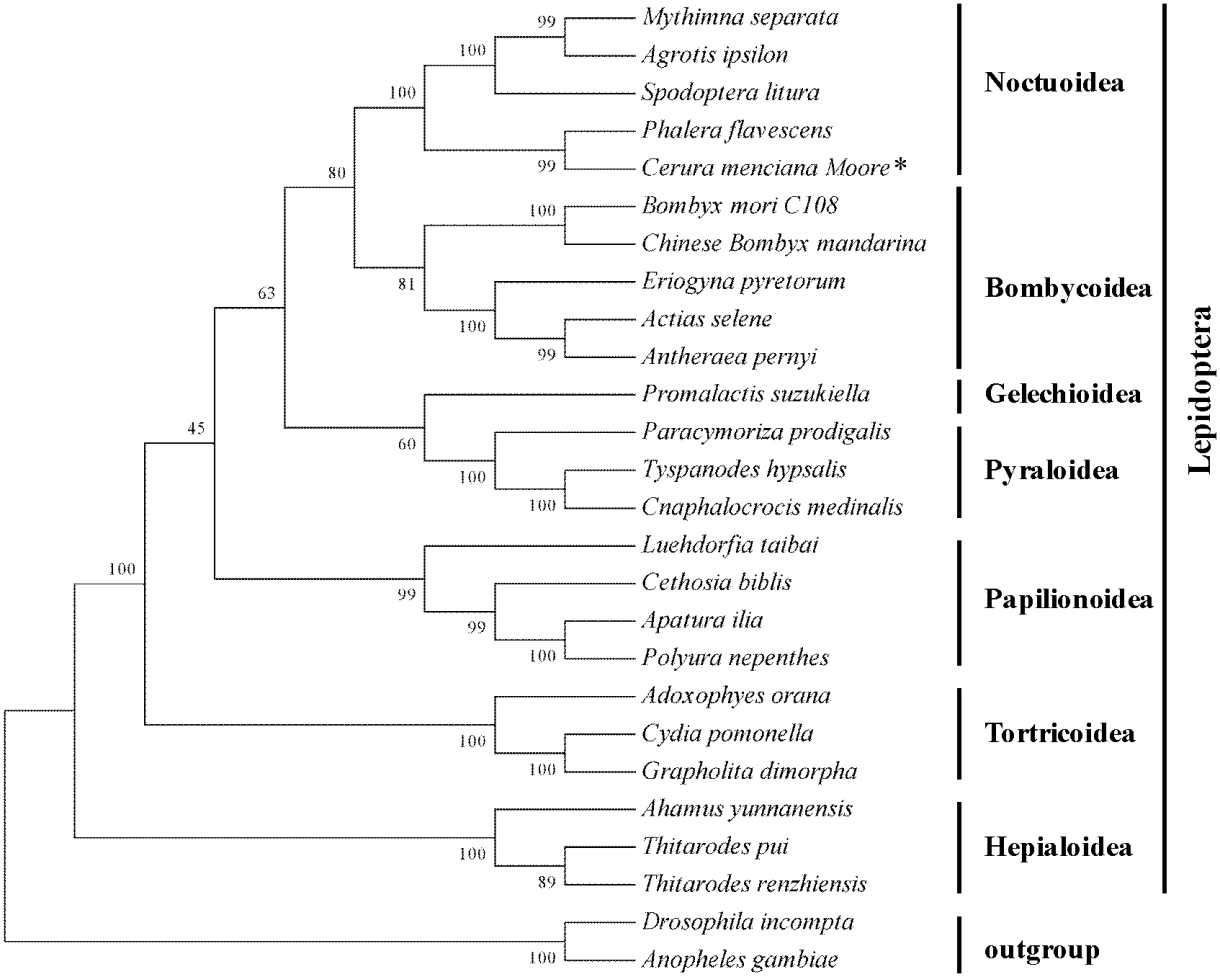
Tree showing the phylogenetic relationships among Lepidopteran insects, constructed using Maximum Likelihood method. Bootstrap values (1000 repetitions) of the branches are indicated. *Drosophila incompta* (NC_025936) and *Anopheles gambiae* (NC_002084) were used as outgroups.

We reconstructed the phylogenetic relationships among the seven superfamilies of lepidopteran using Maximum Likelihood (ML) method based on concatenated nucleotide sequences of the 13 PCGs. The resulting phylogenetic tree revealed that different species from the same family clustered together (Fig 7). The phylogenetic analyses also showed that *C*. *menciana* was most closely related to *P*. *flavescent* of the Notodontidae family. Noctuoidea is closely related to Bombycoidea and Geometroidea, but Hepialoidea was a sister group to the other superfamilies. This result is consistent with that described in previous research [4,36]. Further studies using larger sample sizes are needed to confirm these phylogenetic relationships.
